# The Effect of Temperature on Pressurised Hot Water Extraction of Pharmacologically Important Metabolites as Analysed by UPLC-qTOF-MS and PCA

**DOI:** 10.1155/2014/914759

**Published:** 2014-10-13

**Authors:** B. S. Khoza, L. Chimuka, E. Mukwevho, P. A. Steenkamp, N. E. Madala

**Affiliations:** ^1^Department of Biochemistry, University of Johannesburg, P.O. Box 524, Auckland Park 2006, South Africa; ^2^Molecular Sciences Institute, School of Chemistry, University of the Witwatersrand (WITS), P/B 3, Johannesburg 2050, South Africa; ^3^CSIR Biosciences, Natural Products and Agroprocessing Group, Pretoria 0001, South Africa

## Abstract

Metabolite extraction methods have been shown to be a critical consideration for pharmacometabolomics studies and, as such, optimization and development of new extraction methods are crucial. In the current study, an organic solvent-free method, namely, pressurised hot water extraction (PHWE), was used to extract pharmacologically important metabolites from dried *Moringa oleifera* leaves. Here, the temperature of the extraction solvent (pure water) was altered while keeping other factors constant using a homemade PHWE system. Samples extracted at different temperatures (50, 100, and 150°C) were assayed for antioxidant activities and the effect of the temperature on the extraction process was evaluated. The samples were further analysed by mass spectrometry to elucidate their metabolite compositions. Principal component analysis (PCA) evaluation of the UPLC-MS data showed distinctive differential metabolite patterns. Here, temperature changes during PHWE were shown to affect the levels of metabolites with known pharmacological activities, such as chlorogenic acids and flavonoids. Our overall findings suggest that, if not well optimised, the extraction temperature could compromise the “pharmacological potency” of the extracts. The use of MS in combination with PCA was furthermore shown to be an excellent approach to evaluate the quality and content of pharmacologically important extracts.

## 1. Introduction

*Moringa oleifera* is commonly known as the drumstick or horseradish tree, and a wide variety of nutritional and medicinal attributes have been associated with its roots, bark, leaves, and fruits [1]. The leaves of this plant contain high levels of potassium, calcium, phosphorus, iron, vitamins A and D, essential amino acids, and antioxidants (specifically phenolics and flavonoids) [2, 3]. These nutraceutical properties have given* M. oleifera* the moniker of the miracle tree, or the tree of life, and it is increasingly used as a medical plant in various countries, including South Africa.

Metabolic fingerprinting/metabolomics is a field of study that aims to determine the metabolic changes in a biological system brought about by diet, environmental stress, or disease [4]. The most important factor to optimise when determining such changes is the metabolite extraction step, as this can affect the number of metabolites detected, as well as the reproducibility and reliability of the data obtained [4].

Classical extraction techniques which rely on organic solvents have several limitations: they require large volumes of environmentally unfriendly organic solvents [5], are often time-consuming, and have a low extraction yield [6]. Recently, extraction technology has been progressing with new, nonconventional, and simpler ways to prepare samples, such as microwave-assisted extraction (MAE), supercritical fluid extraction (SFE), pressurised liquid extraction (PLE), ultrasound-assisted extraction (UAE), and pressurised hot water extraction (PHWE) [6–9].

The use of supercritical fluids for the extraction of pharmacologically important compounds is starting to gain momentum in the field of metabolomics [9], but, to date, there is no single extraction method suitable for extracting all metabolites at a given time. This is mainly due to fundamental differences in the physicochemical properties of metabolites, such as polarity.

PHWE is one of the methods that have been proposed to overcome this problem, and it has recently received attention as a “green” solvent extraction method, making use of water as a solvent [6, 12]. It exploits a curious property of water: at a certain temperature and applied pressure, the polarity of water becomes similar to that of some common alcohols (methanol and ethanol) [5, 6, 13]. As demonstrated by Teo et al. [6], the PHWE technique can be carried out in four steps. Firstly, elevated temperature and pressure conditions cause the release of solutes from the sample medium; secondly, the extraction fluid enters the sample environment; thirdly, the solutes dissolve into the extraction fluid; and, finally, the solvent is eluted from the extraction cell into collection vials. Although several different factors have been highlighted as crucial to the success of PHWE, temperature control is the most important. This is because changes in temperature affect the physicochemical properties of water and can also cause thermally labile compounds to decompose [6]. Compared to other extraction approaches, PHWE gives a true reflection of what the general public consumes because, contrary to what scientists apply as method of extraction, water is used as extractive medium for daily consumption of medicinal plants such as* Moringa.*

In this pilot study, PHWE was optimised for* M. oleifera* by adjusting the temperature of the water. The differences in metabolite output at different temperatures were profiled using UPLC-MS in combination with multivariate data models (principal component analysis, PCA). As previously stated, to achieve our main objective, we opted for a plant with known pharmacological metabolites. Thus by monitoring changes of such metabolites due to temperature differences we can hypothesize on the effect of temperature on the pharmacological activity. Our findings suggest that water can successfully be used for the extraction of* M. oleifera* metabolites with pharmacological relevance. However, if not well optimised, temperature affects the metabolite composition of the PHW extracts and the pharmacological activity thereof.

## 2. Materials and Methods

### 2.1. Metabolite Extraction

*M. oleifera* leaves were collected from the Moringa farm (Lebowakgomo, Limpopo, South Africa) and air-dried in a covered area for at least three days. Voucher herbarium specimens (with voucher number NEM001) were prepared and deposited to the Department of Botany, University of Johannesburg. The dried leaves were homogenised to a powder using a pestle and mortar. Metabolites from crushed leaves were extracted using a home-made PHWE instrument. The instrument's stainless steel tubing has the following dimensions: 1/16 in outer dimension (OD) and 0.18 mm inner dimension (ID) connecting the HPLC pump and the extraction cell made from an old HPLC column (150 mm in length and approximately 10 mL) [12]. For the extraction, 600 mg of crushed leaves was mixed with 400 mg of diatomaceous earth (Sigma, USA). The mixture was placed in a stainless steel extraction chamber sealed with steel frits at both ends. The extraction chamber was placed in a refurbished GC oven equipped with a temperature control unit (±1°C) set at the desired temperature (50, 100, or 150°C). Preheated ultra-pure water was pumped through the stainless steel extraction cells inside the oven using a Waters 6000 fluid controller HPLC pump (Waters, MA, USA), until a total volume of 6 mL was collected. During the extraction, the pressure reading on the pump was regulated using a needle back-pressure regulator (Swagelok, Johannesburg, RSA) to maintain 1000 ± 200 psi at a constant flow rate of 2 mL/min. Two (2) mL of PHW extracts were filtered through a 0.22 *μ*m nylon filter and stored at −20°C prior UPLC-qTOF-MS analysis.

### 2.2. Antioxidant Activities

To measure the antioxidant activity of the PHW extracts at different temperatures, the extracts were freeze-dried overnight and reconstituted in 100% methanol to a final concentration of 0.5 mg/mL. For antioxidant activity, 60 *μ*L of 20 *μ*g/mL and 4 *μ*g/mL were mixed with 190 *μ*L of 2,2-diphenyl-1-picrylhydrazyl (DPPH) (90 *μ*M) in methanol. For the control, 60 *μ*L of methanol (in place of extracts) was mixed with 190 *μ*L of DPPH (90 *μ*M) in methanol. The mixture was incubated for 30 min at room temperature (25°C) in total darkness. The total antioxidant activity was read using a multiwell spectrophotometer (BioTek Synergy HT) by measuring the absorbance depletion of DPPH at 515 nm. The percentage DPPH inhibition was determined using the following formula:

(1) %  DPPH  inhibition   =[1−(Absorbance  of  the  extractsAbsorbance  of  control)]×100.

### 2.3. Ultra-Performance Liquid Chromatography

To evaluate the metabolite composition of the PHW extracts, 5 *μ*L of the PHW extracts was analysed on a Synapt UHPLC-high definition MS instrument (Waters, MA, USA) equipped with an Acquity BEH C18 column (100 mm × 2.1 mm with particle size of 1.7 *μ*m) (Waters, MA, USA). Three technical replicates from three independent extract replicates were analysed on the UPLC-MS, resulting in nine replicates per temperature set-point. The composition of mobile phase A consisted of 0.1% formic acid in deionized water, and mobile phase B consisted of 0.1% formic acid in methanol. The column was eluted with a linear gradient at a constant flow rate of 400 *μ*L/min of 5% B over 0.0–2.0 min, 5–95% B over 2.0–22.0 min, held constant at 95% B over 22.0–25.0 min, 95-5% B over 25.0–27.0 min, and a final wash and reequilibration at 5% B over 27–30 min.

### 2.4. Mass Spectrometry Acquisition Parameters

For the MS data acquisition, data was collected in both negative and positive polarity electrospray ionization (ESI) modes for* m/z *range: 100–1000, scan time: 0.1 sec, interscan delay: 0.02 sec with leucine enkephalin (556.3 *μ*g/mL) as a lock mass standard at flow rate: 0.4–1 mL/min, and mass accuracy window of 0.5 Da. The instrument settings were as follows: collision energy of 3 eV, capillary voltage of 2.5 kV, sample cone voltage of 17 V, extraction cone voltage of 5.0 V, MCP detector voltage of 1600 V, source temperature at 120°C, desolvation temperature at 50°C, cone gas flow at 50 (l/h), and desolvation gas flow at 700 (l/h). To achieve metabolite fragmentation patterns necessary for annotation or identification, the collision energy during MS acquisition was experimentally changed by acquiring data at 3 eV, 10 eV, 20 eV, and 30 eV.

### 2.5. Multivariate Data Analyses

To create a data matrix for multivariate data modelling (PCA), UPLC-MS raw data was exported and analysed using Markerlynx XS software (Waters, MA, USA). The analyses were carried out using different parameters and for maximum data output. The following parameters were chosen: retention time (Rt) of 1–27 min, mass range of 100–1000 Da, mass tolerance of 0.02 Da, and Rt window of 0.2 min. The analyses conditions/parameters were kept constant for both negative and positive data. The final data matrix was imported to SIMCA-P software version 12.0 (Umetrics, Sweden) in order to perform PCA. Unless stated otherwise, all PCA models were* pareto *scaled. From the PCA loadings plot, metabolites of which the levels were affected by temperature during extraction were selected. Using the* m/z* of these metabolites, their respective extracted ion chromatograms were generated. The molecular formulae of these ions were computed and selected on the basis that they are within a 5 ppm mass accuracy range. The identities of the ions were searched using the Dictionary of Natural Products database.

## 3. Results and Discussion

Metabolomics studies include the following fundamental steps: sample collection, extraction, analysis of the extracts, data reduction, and statistics [9]. It is important that each step is optimised for maximum data output. We have previously reported on different approaches for method validation to maximise data output, such as chromatographic separations [14] and mass spectroscopic acquisition [15]. In both cases we noted that failure to optimise the above will negatively affect data output and subsequent biological explanations. However, researchers often ignore the importance of the extraction step, with different protocols being applied without validation [9].

As previously stated, there is no single extraction technique able to extract all the metabolites from an organism, but attention has been paid to developing such a technique by means of optimising existing protocols. Over the decades, it has been accepted as the rule of thumb that choice of solvent is the main factor in achieving maximum metabolites extraction [9]. However, it is not only the interaction between solvents and solutes that results in maximum metabolite extraction but other factors, such as “nondestructive” mechanical forces aimed at dissociation of the analyte from the matrix, which may also play a role [16].

**Figure 1:**
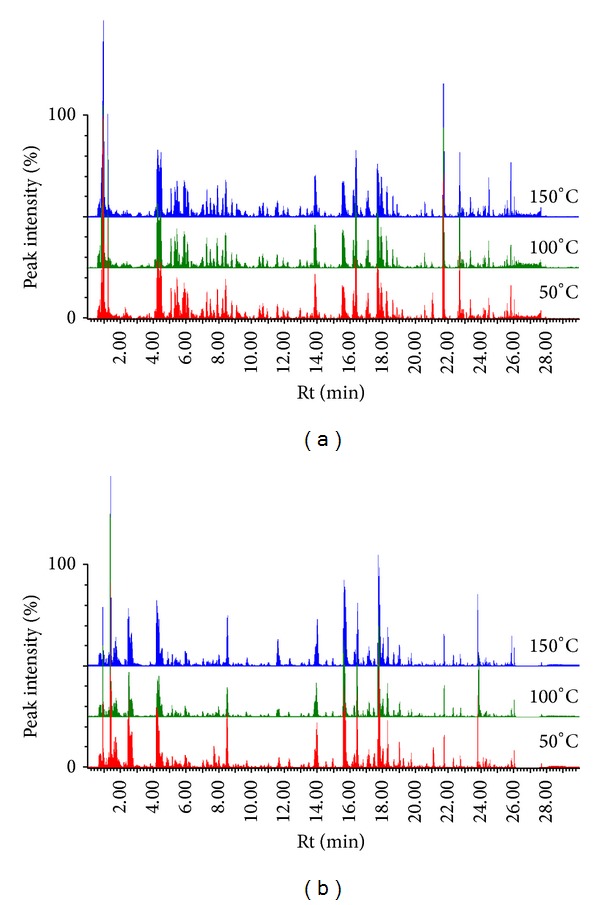
Representative base peak intensity (BPI) of UHPLC-qTOF-MS chromatograms of PHW extracts at different temperatures. The samples were analysed at different MS ionisation modes; ESI− (a) and ESI+ (b).

This study emphasises the fact that it is important to choose an extraction technique aligned to the main objective of the study. For instance, in ethnopharmacological studies scientists often apply very sophisticated methods for metabolite extraction, neglecting the fact that the layman (usually traditional healers) does not have access to such methods. Here, we present a pilot study that will form the basis of a bigger investigation to prove the scientific efficacy of* M. oleifera* plant extracts for medical and nutritional supplements. In South Africa, traditional healers suggest that* M. oleifera* extraction can be achieved by means of cooking, boiling, or mixing with warm water. To mimic these traditional medicinal uses of* Moringa*, we chose PHWE as a method of extraction. Here, the temperature of the solvent (water) was intentionally altered to evaluate the effect thereof on the extracted metabolite composition. Following a visual inspection of the UPLC-MS chromatograms, we concluded that PHWE can be used successfully for the extraction of metabolites from* M. oleifera* (Figure 1). Visually there are, however, no significant differences between extracts generated at different temperatures.

**Figure 2:**
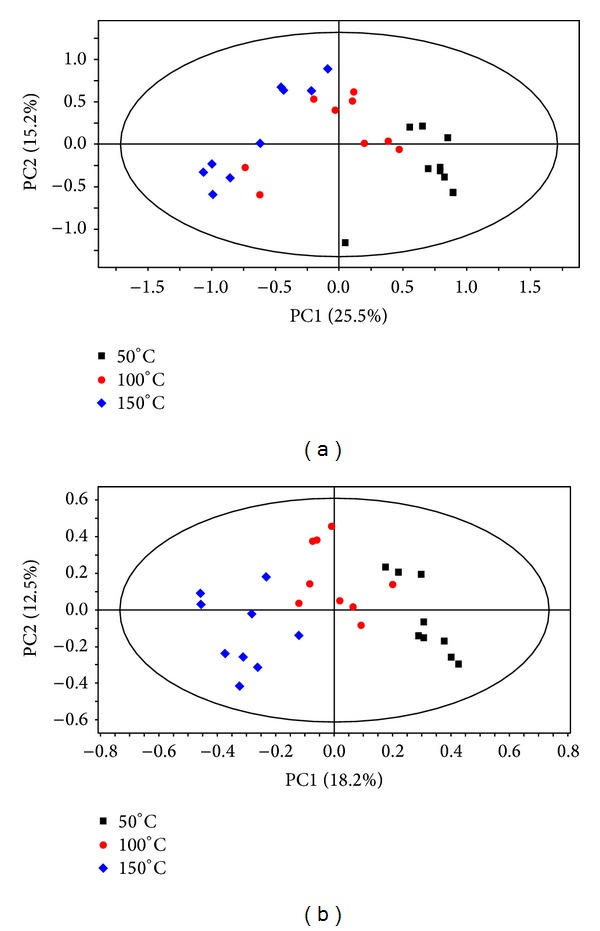
PCA score plots showing the different clustering and separation of* Moringa* samples extracted at different temperatures and analysed by UPLC-qTOF-MS on ESI− (a) and ESI+ (b).

Due to the large volumes of data generated in metabolomics studies, several mathematical models have been developed to fully unearth the hidden elements from the data. One of these techniques is PCA [17]. By definition, PCA is mathematically defined as an orthogonal linear transformation of possibly correlated variables into a smaller number of uncorrelated variables called principal components, in which the greatest variance within the data by any projection is explained on the first coordinate (called the first principal component), and the less variance is explained/projected by subsequent principal components [18]. In the current study, PCA was used to classify PHW extracts generated using different temperatures based on the metabolite composition. As can be seen from Figures 2(a) and 2(b), PCA score plots revealed differential clusteringof samples into distinctive groups. From this it can be concluded that samples group together, due to similarities in their chemical composition. Thus, samples extracted using the same temperature grouped together due to similarities in their chemical composition (Figures 2(a) and 2(b)). Originally, four different temperatures (50, 100, 150, and 200°C) were chosen, but at 200°C samples proved to be unstable. Only the first three temperature variations were thus used. Using PCA score plots (Figures 2(a) and 2(b)), there is a definite underlying trend with samples grouping in a definitive sequence.

From the PCA loadings plots there were no distinctive patterns; however, most of the metabolites levels seemed to increase with an increase in temperature. From the results, Table 1, it can be seen that, at 50°C, there were few chlorogenic acids (CGA) and flavonoids which were efficiently extracted while structurally similar metabolites were found to increase with an increase in temperature, thus, at 150°C. Clearly, temperature effects on metabolite concentration cannot be predicted; hence the importance of optimising the extraction process prior to any major experiments should be mandatory.

The antioxidant activity of the extracts was also studied by monitoring the DPPH scavenging potency of the extracts at different temperatures. It can be noted that the antioxidant capacity decreases with an increase in temperature (Figure 4). To fully explain this trend, the metabolites that caused the separation between groups on the PCA models were evaluated for both ESI (+) and ESI (−). The results show that due to the nature of the affected metabolites, such as chlorogenates and flavonoids (Table 1), the results of PCA (Figure 2) and those of antioxidant activity (Figure 4) can be positively correlated.

**Figure 3:**
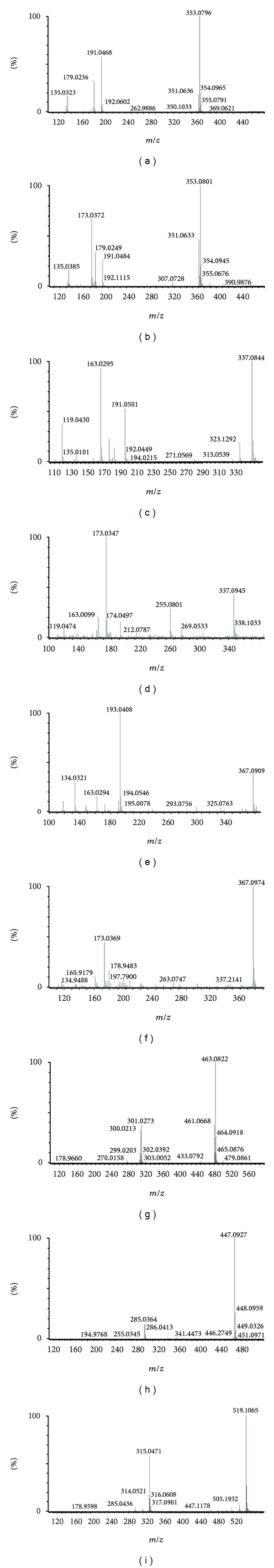
The representative UPLC-QTOF-MS spectra showing fragmentation patterns of 3CQA (a), 4CQA (b), 3pCoQA (c), 4pCoQA (d), 3FQA (e), 4FQA (f), quercetin-*O-*glycoside (g), kaempferol-*O-*glycoside (h), and isorhamnetin-*O-*glycoside (i) generated at a collision energy of 30 eV.

**Figure 4:**
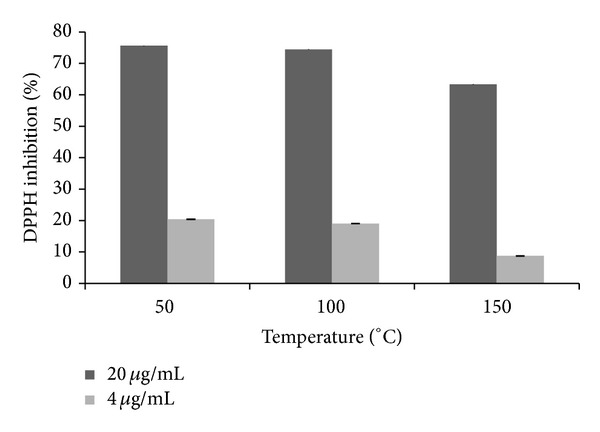
Antioxidant activities, based on DPPH radical scavenging potential, of* Moringa *extracts generated at different temperatures.

Furthermore, PCA loading plots were evaluated to identify metabolites responsible for the differential grouping seen on the PCA score plot (data not shown). We tentatively identified the metabolites responsible for the clustering using the Dictionary of Natural Products (DNP) in an approach we reported previously [19] and by comparing MS fragmentation patterns already reported in the literature [10, 11] (Figure 3). The putatively identified metabolites provided a snapshot of the effect of temperature on the extraction of important metabolites. Here it can be noted that levels of polyphenolic metabolites such as CGA and flavonoids were affected by temperature changes (Figure 3 and Table 1). Briefly, as previously stated, metabolite identification was achieved through the use of online databases and comparison with previously published data. For identification of chlorogenic acids, MS settings were sequentially altered until a stable fragmentation characterised by the formation of C1 [caffeic acid-H], C2 [caffeic acid-CO_2_], Q1 [quinic acid-H], and Q2 [quinic acid-H2O] ions [11]. These ions have been used previously to accurately annotate the different isomers of CGA (Figure 3). A similar but different approach was followed for the identification of flavonoids molecules. For flavonoids, molecular formula of the pseudomolecular ions ([M-H]^−^) representing quercetin (*m/z* 300/301), kaempferol (*m/z* 284/285), and isorhamnetin (*m/z* 314/315) was computed. Due to advances in mass spectrometry such as approaches described herein, flavonoids and other polyphenolic identifications have become easier. During ESI negative, flavonoid fragmentation patterns can be predicted without the use of authentic standards. For instance, the removal of* O-*glycosylated sugar can happen through either heterolytic cleavage with hydrogen migration or hemolytic sugar cleavage to produce an aglycone product ion Y_0_^−^ or an aglycone radical ion [Y_0_-H]^−^, respectively [20]. In the past, the ratio of [Y_0_-H]^−^/  Y_0_^−^ has been used with great success in determining the original sugar position before fragmentation [21]. Thus, by comparing mass spectra generated at different collision energy levels, the position and, occasionally, the type of sugar can be deduced. Overall, in the current study, this information was used in combination with comparison to previously published data to putatively identify metabolites.

**Figure 5:**
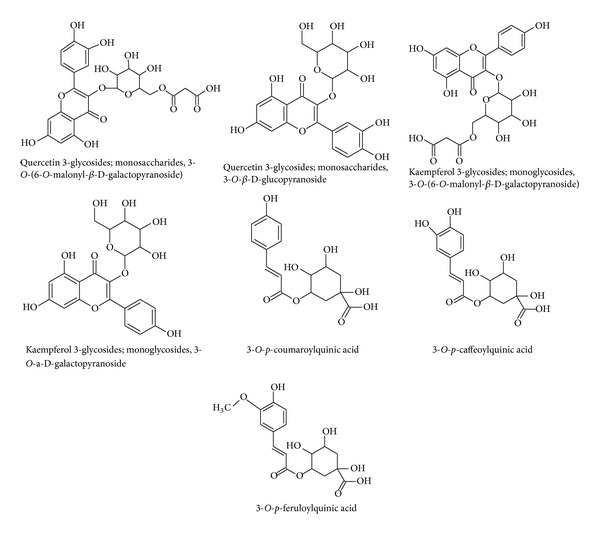
List of tentatively identified metabolites previously reported to have pharmacological activities [10, 11].

From the literature,* M. oleifera* has several bioactive compounds, with glucosinolates, flavonoids, and phenolic acids being the most common [10, 22, 23]. As noted above CGAs and flavonoids were amongst the dominant in the chromatograms, suggesting that PHWE is good for polyphenol extraction. The same metabolites have also been shown to make up a large part of the* Moringa *metabolite profile [10] and have several biological activities. For instance, CGAs have been shown to play a significant role during glucose metabolism [24]. Using the study by Bennett et al. [10] as the template, metabolites with known pharmacological activities were also found to be extracted by PHWE (Figure 5). These include but are not limited to CGAs and flavonoids. These metabolites were previously extracted by organic solvents such as methanol [11] and the fact that the same metabolites can be extracted by water is an indication that PHWE is a superior and comparable extraction method. In most cases, to prove the feasibility and legitimacy of this method, another traditional solvent extraction method is used. In the current study, no alternative method was used, but the results were comparable to those found by Bennett et al. [10], in which ethanol was used to extract CGAs and flavonoids.

In several studies, CGAs have been shown to affect sugar metabolism by inhibiting glucose-6-phosphate translocase, an enzyme responsible for converting glycogen in the liver to glucose [25]. Apart from the above, CGA and flavonoids have been shown to have health benefits. In particular, a diet containing CGAs has been shown to have antioxidant, anti-inflammatory, antiviral, and anticancer properties [26–31]. Thus, any extraction technique that maximises the extraction of such metabolites can be regarded as superior and applicable to pharmacological studies. From the current study then, PHWE can be said to be an ideal method for the extraction of metabolites of medicinal importance. The use of abundant and environmentally friendly water as the principal solvent and the fact that it is a similar extraction process to those used by most traditional health practitioners make it appealing and directly comparable to traditional methods still in use.

## 4. Conclusion

Using other techniques, including microwave digestion, the effect of temperature on metabolite extraction from plants has been studied with mixed outcomes. It has been noted that although temperature has some positive influence (such as better interactions between the solvent and the active sites of the solute [32]), temperature effects, as reaffirmed by our results, are not always predictable [33, 34]. The results of this present study suggest that if temperature is to be relied upon for extraction, major optimisation should be done to prevent undesirable outcomes, such as a loss of active ingredients from the extracts. Though it was not the main objective of the current study, PHWE has been identified as a suitable method to extract metabolites for metabolomics-based studies, because of its ability to extract a wide spectrum of secondary metabolites with high reproducibility. Furthermore, some of the metabolites extracted here using PHWE were previously shown to have beneficial health properties, making PHWE a suitable metabolite extraction technique for pharmacological studies. The combination of UPLC-MS and PCA can be used to optimise extraction methods as both provide a holistic view of the underlying metabolite patterns. Although not part of the current study, it can be seen that* M. oleifera* contains diverse metabolites with known medicinal properties and the results of the current study thus warrant further investigations into the pharmacological activities of* M. oleifera *extracts.

## Figures and Tables

**Table 1:** List of putatively annotated pharmacologically relevant metabolites extracted from *M. oleifera* using the PHWE technique.

Compound name	Rt (min)	[M-H]^−^	Molecular formula
3-Caffeoylquinic acid (3-CQA)	4.31	353.0853	C_16_H_18_O_9_*
4-Caffeoylquinic acid (4-CQA)	5.93	353.0859	C_16_H_18_O_9_*
3-c*is*-*p*-Coumaroylquinic acid (3-*p*CoQA)	5.39	337.0896	C_16_H_18_O_8_**
3-*trans*-*p*-Coumaroylquinic acid (3-*p*CoQA)	5.50	337.0899	C_16_H_18_O_8_**
4-*cis*-*p*-Coumaroylquinic acid (4-*p*CoQA)	7.67	337.0905	C_16_H_18_O_8_**
4-*trans*-*p*-Coumaroylquinic acid (4-*p*CoQA)	7.75	337.0931	C_16_H_18_O_8_**
3-*cis*-Feruloylquinic acid (3-FQA)	6.13	367.1012	C_17_H_20_O_9_
3-*trans*-Feruloylquinic acid (3-FQA)	6.38	367.10428	C_17_H_20_O_9_
4-Feruloylquinic acid (4-FQA)	9.30	367.1048	C_17_H_20_O_9_
Caffeic acid	5.88	179.0331	C_9_H_8_O_4_*
Quinic acid (QA)	0.96	191.0165	C_7_H_12_O_6_
Apigenin-7-glycoside	8.46	593.1523	C_27_H_30_O_15_
Quercetin 3-glycosides; monosaccharides, 3-O-(6-O-malonyl-*β*-D-galactopyranoside)	17.49	549.2561	C_24_H_22_O_15_**
Quercetin 3-glycosides; monosaccharides, 3-O-*β*-D-glucopyranoside	13.91	463.0798	C_21_H_20_O_12_**
Kaempferol 3-glycosides; monoglycosides, 3-O-(6-O-malonyl-*β*-D-galactopyranoside)	18.64	533.0915	C_24_H_22_O_14_*
Kaempferol 3-glycosides; monoglycosides, 3-O-a-D-galactopyranoside	17.02	447.0840	C_21_H_20_O_11_**
Quercetin 3-glycosides; monosaccharides, 3-*O*-[4-carboxy-3-hydroxy-3-methylbutanoyl-(→6)-*β*-D-galactopyranoside]	16.27	607.1305	C_27_H_28_O_16_**
Kaempferol	21.06	285.0410	C_15_H_10_O_6_*
Isorhamnetin 3-glycosides; monosaccharides, 3-*O*-(6-*O*-acetyl-*β*-D-glucopyranoside)	18.29	519.1061	C_24_H_24_O_13_**
Quercetin 3-glycosides; monosaccharides, 3-O-(6-O-acetyl-a-D- glucopyranose)	17.18	533.1090	C_23_H_22_O_14_**

*Extracted more with 50°C.

∗∗Extracted more with 150°C.
